# The association between cumulative radiation dose and the incidence of severe oral mucositis in head and neck cancers during radiotherapy

**DOI:** 10.1002/cnr2.1317

**Published:** 2020-12-09

**Authors:** Tomiko Sunaga, Akiko Nagatani, Naokazu Fujii, Touji Hashimoto, Toru Watanabe, Tadanori Sasaki

**Affiliations:** ^1^ Department of Hospital Pharmaceutics, School of Pharmacy Showa University Tokyo Japan; ^2^ Department of Pharmacy Showa University Fujigaoka Hospital Yokohama Japan; ^3^ Department of Otorhinolaryngology Showa University Toyosu Hospital Tokyo Japan; ^4^ Department of Radiology Showa University Fujigaoka Hospital Yokohama Japan

**Keywords:** concurrent chemotherapy, cumulative radiation dose, head and neck cancer, oral mucositis, predictive factors, radiotherapy

## Abstract

**Background:**

Quality of life can be influenced by oral mucositis (OM), and it is necessary to implement OM management strategies before the initiation of radiotherapy (RT) in patients with head and neck cancer (HNC).

**Aims:**

To examine the association between the cumulative radiation dose and the incidence of severe OM in HNC patients receiving RT.

**Methods and results:**

A retrospective observational cohort study was conducted in a Showa University Fujigaoka Hospital, in Japan. We retrospectively analyzed 94 patients with HNC who developed OM during RT. We defined OM as a more than grade 2 OM. The cumulative incidence of OM curves of the two categories was estimated using the Kaplan–Meier method and compared using the log‐rank test. We estimated the hazard ratio (HR) for OM after the adjustment of factors for covariates using Cox's regression analysis. Patients with smoking history had a significantly later development of OM than those with no smoking history (20 Gy‐incidence OM 68.7% vs 39.7%, *P* = .003). In contrast, patients undergoing concurrent chemotherapy had an earlier development of OM than those undergoing RT alone (20 Gy‐incidence OM 24.2% vs 55.7%, *P* < .001). Multivariate analysis revealed that no smoking history and concurrent chemotherapy were independent predictive factors, with a HR of 0.526 (*P* = .025) and 2.690 (*P* < .001), respectively.

**Conclusion:**

We demonstrated that no smoking history and concurrent chemotherapy may be predictive of OM in HNC patients.

## 1 INTRODUCTION

Head and neck cancers (HNC) represent 5% of all cancers. In 2018, they accounted for an estimated 887 649 new cancer cases and 453 307 cancer‐related deaths globally.^1^ The head and neck are also closely related to swallowing, voice respiration, articulation, and mastication, and the loss of these functions can dramatically lower patients' quality of life (QOL). Kam et al reported that the incidence of suicide in patients with HNC is more than thrice that of the general U.S. population.^2^ They also indicated that this may be linked to the relationship between anatomic sites and the ability to speak and/or swallow. The standard treatment of HNC is radiotherapy (RT), to keep these functions. In advanced HNC, chemotherapy is concurrently administered with RT. Sarraf et al indicated that chemoradiotherapy (CRT) is superior to RT alone for patients with advanced nasopharyngeal cancers for progression‐free survival and overall survival (OS).^3^

Radiation‐induced oral mucositis (OM) is common among patients with HNC and is the most debilitating side effect of RT.^4^ CRT increases the incidence of side effects compared to RT alone; Hata et al reported that OM risk of more than grade 2 increases by 5.6 times compared to RT alone.^5^ OM leads to reduced oral intake and increases dysphagia due to pain, which can dramatically lower the patients' QOL. Chen et al revealed that OM was the most common oral dysfunction.^6^ Therefore, it is important to complete treatment to maintain the QOL of patients while properly managing pain control.

It is necessary to implement OM management strategies before the initiation of RT in patients with HNC.^7^ Some studies have reported on the risk factors for OM in HNC patients.^8^, ^9^, ^10^ The initial clinical signs of OM include mucosal erythema and superficial sloughing that may occur with a cumulative radiation dose of 20 to 30 Gy, which is accompanied by the beginning of the breakdown of the intact mucosa followed by ulceration.^11^ Vera et al, also reported that HNC patients with nasopharyngeal or oropharyngeal tumors who receive cumulative radiation doses >50 Gy are more likely to develop OM.^10^ However, there is no evidence on the predictive factors for OM in relation to cumulative radiation dose. It is important to predict OM before the initiation of RT in patients with HNC. Therefore, we conducted this retrospective analysis of patients with HNC who were treated with RT to investigate the relationship between cumulative radiation dose and the incidence of severe OM.

## 2 METHODS

We conducted a retrospective cohort study using data obtained from medical records. This retrospective study included HNC patients who were admitted or attended to the Showa University Fujigaoka Hospital between January 2005 and March 2015. The inclusion criteria were as follows: participants experienced OM during RT with HNC patients. Participants who experienced no OM were excluded due to investigation of the relationship between cumulative radiation dose and the incidence of severe OM. We were defined incident of OM as a more than grade 2 OM due to severe OM lower the patients' QOL. Therefore, 94 patients were eligible for the analysis. We evaluated the association between cumulative radiation dose and the incidence of OM in HNC patients.

The tumors were histologically diagnosed and staged according to the TNM classification; they were confirmed by neck and chest computed tomography, bone scintigraphy, endoscopy, and histological diagnosis by biopsy. We extracted the population using the diagnosis code in our original system.

The ethics committee of our institution approved the study (approval number: 201516).

### 2.1 Treatment

Patients were irradiated with standard radiation (total 35 counts, 2 Gy once a day) or hyperfractionation (total about 58 counts, 1.2 Gy twice a day). The treatment period was about 6 to 8 weeks. Patients who received concurrent chemotherapy were administered with oral tegafur/gimeracil/oteracil (S‐1) (80 mg/m^2^),^12^ cetuximab (first; 400 mg/m^2^, second; 250 mg/m^2^)^13^ or, S‐1 and nedaplatin (SN) therapy (S‐1; 80 mg/m^2^, Nedaplatin 90 mg/m^2^).^14^

### 2.2 Evaluation of OM

OM was graded as 1‐4 according to the National Cancer Institute Common Toxicity Criteria for Adverse Events, version 4.0.^15^ The most severe grade of OM was based on the worst OM from the first to the last day of RT. We also investigated the time of onset of OM and the time of the worst severity of OM.

### 2.3 Collected variables

Three researchers collected the data. Baseline characteristics, including patient demographic data (age and sex), alcohol history, smoking history, type of radiation therapy (standard and hyperfractionation), stage, concurrent chemotherapy, and biological parameters (prior white blood cell level [WBC], prior alanine aminotransferase level [ALT], prior creatinine level [Cr], and prior albumin level [Alb]).

### 2.4 Statistical methods

We evaluated the association between cumulative radiation dose and the incidence of OM. When we analyzed factors with a frequency of 65% in 94 patients, we could evaluate a hazard ratio of 1.85, with a power of 80%.

We classified OM into three grades: grades 1, 2, and 3. We observed the following factors and classified them into two categories: age (<65 years vs ≥65 years), sex (male vs female), alcohol history (absent vs present), smoking history (absent vs present), type of RT (standard vs hyperfractionation), stage (1 and 2 vs 3 and 4), chemotherapy (absent vs concurrent), prior WBC level (<4000/μL vs ≥4000/μL), prior ALT level (<50 IU/L vs ≥50 IU/L), prior Cr level (<1.00 mg/dL vs ≥1.00 mg/dL), and prior Alb level (<3.5 g/dL vs ≥3.5 g/dL). The cumulative incidence of OM curves using two or three categories was estimated using the Kaplan‐Meier method and compared by the log‐rank test. The cumulative incidence of OM was defined as the incidence of more than grade 2 OM in this study. We estimated the hazard ratios (HRs) for the incidence of OM after the adjustment for covariates using Cox's regression analysis proportional hazards model (stepwise methods). The time of onset of OM (day) and the cumulative radiation dose (Gy) were compared using the Student's *t* test. *P*‐values less than .05 were considered statistically significant. The statistical analyses were performed using IBM SPSS Statistics 23.

## 3 RESULTS

Baseline characteristics are summarized in Table 1. The mean age of all patients was 67.6 ± 10.2 years, and 92.6% of the patients were male. Smoking and alcohol history were positive in 83.0% and 83.0% of the patients, respectively. Concurrent chemotherapy was administered in 64.9% of the patients. The mean cumulative radiation dose was 68.1 ± 4.5 Gy. The grades of OM were 1 in 17 patients (18.1%), 2 in 65 patients (69.1%), and 3 in 12 patients (12.8%). The time of onset and the worst severity of OM were 15.0 ± 9.5 days and 26.5 ± 13.0 days, respectively. The time of onset of OM was significantly shorter in patients undergoing concurrent chemotherapy than those undergoing RT alone (11.3 ± 6.6 days vs 21.8 ± 10.2 days, *P* < .001). The onset times of grades 1, 2, and 3 of OM were 24.6 ± 11.1 days, 14.1 ± 8.0 days, and 7.6 ± 3.9 days, respectively.

**TABLE 1:** Patient characteristics (n = 94)

Variables	n (%), M ± SD	
Age (years)	67.6 ± 10.2	
Sex		
Male	87	(92.6)
Female	7	(7.4)
Alcohol history		
Absent	16	(17.0)
Present	78	(83.0)
Smoking history		
Absent	16	(17.0)
Present	78	(83.0)
Type of radiation therapy		
Standard	55	(58.5)
Hyperfractionation	39	(41.5)
Cumulative radiation dose (Gy)	68.1 ± 4.5	
Primary tumor location		
Paranasal sinuses/nasopharynx/oral cavity	4	(4.2)
Oropharynx	28	(29.8)
Hypopharynx/larynx	62	(66.0)
Stage		
I	28	(29.8)
II	18	(19.1)
III	13	(13.8)
IV	35	(37.2)
Chemotherapy		
Absent	33	(35.1)
Concurrent	61	(64.9)
S‐1 + Nedaplatin	45	(73.8)
Cetuximab	4	(6.5)
S‐1	12	(19.7)
WBC (×10^3^/μL)	6.6 ± 2.7	
ALT (IU/L)	20 ± 12	
Cr (mg/dL)	0.84 ± 0.30	
Alb (g/dL)	4.1 ± 0.4	

Abbreviation: S‐1, tegafur/gimeracil/oteracil.

**FIGURE 1:**
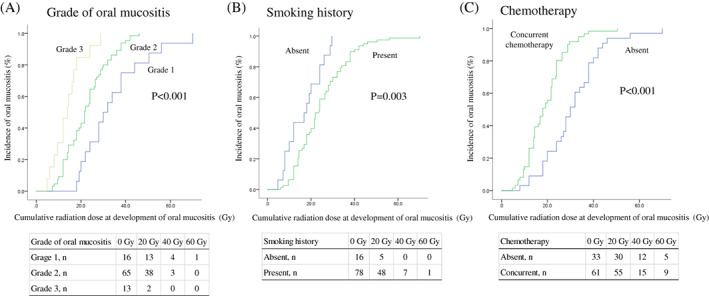
Kaplan‐Meier the incidence of oral mucositis curves according to the grade of oral mucositis, smoking history, and chemotherapy

Univariate analysis revealed an earlier development of OM in patients who experienced more severe OM during RT for HNC (20 Gy incidence OM 18.7% vs 43.1% vs 84.6%, *P* < .001, Figure 1A). Patients undergoing concurrent chemotherapy also had an earlier development of OM compared with those undergoing RT (20 Gy incidence OM 24.2% vs 55.7%, *P* < .001, Table 2, Figure 1C). In contrast, patients with a history of smoking also had significantly delayed OM compared with patients with no history (20 Gy incidence OM 68.7% vs 39.7%, *P* = .003, Table 2, Figure 1B). Stage 3/4 and hyperfractionation of RT were also significantly associated with the development of OM (Table 2). However, there was no significant association between the development of OM and age, sex, and alcohol history.

**TABLE 2:** Univariate analysis of the relationship between cumulative radiation dose and the incidence of oral mucositis

Variables	Oral mucositis	
	20 Gy (incidence of oral mucositis)	
	(%)	*P* value
Age (years)		.415
<65	40.6	
≥65	46.8	
Sex		.857
Male	47.1	
Female	14.3	
Alcohol history		.202
Absent	25.0	
Present	48.7	
Smoking history		.003^*^
Absent	68.7	
Present	39.7	
Type of radiation therapy		.001^*^
Standard	41.8	
Hyperfractionation	48.7	
Stage		<.001^*^
I, II	34.8	
III, IV	54.2	
Chemotherapy		<.001^*^
Absent	24.2	
Concurrent	55.7	
WBC (×10^3^/μL)		.367
<4.0	50.0	
≥4.0	43.9	
ALT (IU/L)		.819
<50	44.4	
≥50	50.0	
Cr (mg/dL)		.373
<1.00	44.9	
≥1.00	43.7	
Alb (g/dL)		.869
< 3.5	25.0	
≥ 3.5	45.6	

^*^ *P <* .05.

Smoking history and concurrent chemotherapy were predictive of the development of OM on multivariate analysis (Table 3). The HR for smoking to no smoking history was 0.526 (95% confidence interval [CI], 0.300‐0.922; *P* = .025), and the HR for concurrent chemotherapy to RT alone was 2.690 (95% CI, 1.691‐4.279; *P* < .001).

**TABLE 3:** Multivariate analysis of the relationship between cumulative radiation dose and the incidence of oral mucositis

Variables	*β*	HR	(95% CI)	*P* value
Smoking history				
Absent vs present	−0.643	0.526	(0.300‐0.922)	.025
Chemotherapy				
Absent vs concurrent	0.990	2.690	(1.691‐4.279)	<.001

Abbreviations: CI, confidence interval; HR, hazard ratio = exp(*β*).

## 4 DISCUSSION

We found that severe OM developed significantly earlier in HNC patients who had concurrent chemotherapy and had no smoking history. To the best of our knowledge, this is the first study to investigate these relationships in patients with HNC receiving RT. In our study, we demonstrated that a positive smoking history was associated with a 0.526‐fold increase in the incidence of OM in patients with HNC. We also indicated that patients with HNC and concurrent chemotherapy had a 2.690‐fold increase in the incidence of OM than patients with HNC and RT alone. Therefore, healthcare providers should be conscious of OM development during RT in patients with HNC, especially in those who received concurrent chemotherapy and have no smoking history. Management strategies should be implemented before the initiation of RT accordingly.

The initial clinical signs of OM, including mucosal erythema and superficial sloughing, may occur when intact mucosa begins to break down following a cumulative radiation dose of 20 to 30 Gy; this is followed by ulceration.^11^ Vera et al reported that HNC patients with nasopharyngeal or oropharyngeal tumors who receive cumulative radiation doses >50 Gy are more likely to develop OM.^10^ However, several patients developed OM with less than 40 Gy in this study. OM developed significantly earlier in patients who experienced its more severe forms during RT for HNC. In addition, the time of onset of OM was also observed earlier in patients who had grade 3 OM. Healthcare providers should exercise caution with OM management and monitoring in the aforementioned groups of patients as OM may develop early.

We previously demonstrated that concurrent chemotherapy was identified as a significant, independent risk factor for the severity of OM.^16^ Hata et al reported that 5‐FU was related to worse OM.^5^ This study also revealed a relationship between cumulative radiation dose, even lower doses, and the development of OM in patients with HNC during RT. Therefore, healthcare providers should be strategic with OM management during concurrent chemotherapy, even when the cumulative radiation dose is low.

Jyoti et al reported that former and active tobacco smoking during RT for cervical cancer is associated with unfavorable disease‐free survival and OS outcomes.^17^ Chen et al also reported that tobacco smoking during RT for HNC is associated with unfavorable outcomes.^18^ Hemoglobin binds to nitric oxide (NO), oxygen (O_2_), and carbon monoxide (CO). Therefore, when NO in smoke binds to hemoglobin, O_2_ carrying capacity is affected, and the partial pressure of O_2_ in cancer lesions is reduced; this, in turn, reduces the effectiveness of RT. In our study, we found that patients with no smoking history significantly had an increased incidence of OM compared to patients with smoking history during RT. The physiological effects of smoking may reduce the effectiveness of RT and prolong the course of OM.

Patients treated with hyperfractionation and accelerated fractionation with concomitant boost had significantly better local‐regional control than those treated with standard fractionation.^19^ However, the standard fractionation resulted in fewer adverse effects compared to hyperfractionation. In our study, although it was consistent that hyperfractionation was more at risk of OM than standard fractionation, this finding was not observed in multivariate analysis. We hypothesized that patients who received concurrent chemotherapy had a higher hyperfractionation rate than that in patients receiving radiation therapy. In addition, although the stage 3 or 4 group had a higher OM risk than those in the stage 1 or 2 groups in univariate analysis, we did not observe this finding in multivariate analysis. Stage may have been a confounding factor as more patients in the stage 1 and 2 groups received radiation therapy compared to those in the stage 3 or 4 groups.

This study had limitations. Our sample size was small and from a single institution. This study was also retrospective, and it was difficult to investigate clinical parameters in detail. In particular, the details of smoking history are important, including the number of cigarettes per day, smoking time, and passive smoking. We did not consider other risks such as preventive oral care and dry mouth, which are related to OM. Although the follow‐up ended with the last RT in this study, we did not consider prognosis in patients with HNC.

In conclusion, we demonstrated that no smoking history and concurrent chemotherapy are predictive factors of severe OM related to cumulative radiation dose. However, smoking history needs to be considered in more detail in the future. OM reduces oral intake and increases dysphagia due to the associated pain and can dramatically lower patients' QOL. Therefore, healthcare providers need to strategize OM management with these considerations. We recommend stringent management and monitoring of patients receiving concurrent chemotherapy, even when the cumulative radiation dose is low because early OM may progress to a severe state. Prospective studies on QOL that investigate QOL benefits related to management strategies may underscore the significance of concurrent chemotherapy and negative smoking history as markers of severe OM that need to be monitored closely by healthcare providers.

## CONFLICT OF INTEREST

The authors have no conflicts of interest to report.

## AUTHORS' CONTRIBUTIONS

Conceptualization, T.S., A.N. and T.W.; Methodology, T.S.; Software, T.S.; Validation, T.S.; Investigation, A.N., N.F. and T.F.; Formal analysis, T.S.; Resources, T.S.; Data curation, T.S.; writing‐original draft, T.S.; Writing‐review & editing, T.S., T.W. and T.S.; Visualization, T.S.; Supervision, T.H. and T.S.; Project administration, T.S. The final version of the paper was seen and approved by all authors.

## ETHICAL STATEMENT

The study was approved by the Ethics Committee at Showa University Fujigaoka Hospital, Japan (approval number 201516). In this retrospective cohort study, informed consent from the patients was not needed according to the ethical approval.

## Data Availability

The data that support the findings of this study are available from the corresponding author upon reasonable request.

## References

[1] Bary F, Ferlay J, Soerjomataram I, Siegel RL, Torre LA, Jemal A. Global cancer statistics 2018: GLOBOCAN estimates of incidence and mortality worldwide for 36 cancers in 185 countries. CA J Clin. 2018;68:394‐424. 10.3322/caac.21492. 30207593

[2] Kam D, Salib A, Gorgy G, et al. Incidence of suicide in patients with head and neck cancer. JAMA Otolaryngol Head Neck Surg. 2015;141:1075‐1081. 10.1001/jamaoto.2015.2480. 26562764

[3] Al‐Sarraf M, LeBlanc M, Giri PG, et al. Chemoradiotherapy versus radiotherapy in patients with advanced nasopharyngeal cancer: phase III randomized intergroup study 0099. J Clin Oncol. 1998;16:1310‐1317. 10.1200/JCO.1998.16.4.1310. 9552031

[4] Trotti A, Bellm LA, Epstein JB, et al. Mucositis incidence, severity, and associated outcomes in patients with head and neck cancer receiving radiotherapy with or without chemotherapy: a systematic literature review. Radiother Oncol. 2003;66:253‐262. 10.1016/s0167-8140(02)00404-8. 12742264

[5] Hata H, Ota Y, Ueno T, et al. Oral mucositis frequency in head and neck chemoradiotherapy. Toukeibugan. 2007;33:48‐53.

[6] Chen SC. Oral dysfunction in patients with head and neck cancer: a systematic review. J Nurs Res. 2019;27:e58. 10.1097/jnr.0000000000000363. 31688276

[7] Kawashita Y, Soutome S, Umeda M, Saito T. Oral management strategies for radiotherapy of head and neck cancer. Jpn Dent Sci Rev. 2020;56:62‐67. 10.1016/j.jdsr.2020.02.001. 32123547 PMC7037635

[8] Bossi P, Numico G, Santis VD, et al. Prevention and treatment of oral mucositis in patients with head and neck cancer treated with (chemo) radiation: report of an Italian survey. Support Care Cancer. 2014;22:1889‐1896. 10.1007/s00520-014-2166-7. 24566870

[9] Takase H, Sakata T, Yamano T, Sueta T, Nomoto S, Nakagawa T. Advantage of early induction of opioid to control pain induced by irradiation in head and neck cancer patients. Auris Nasus Larynx. 2011;38:495‐500. 10.1016/j.anl.2010.12.012. 21277720

[10] Vera‐Llonch M, Oster G, Hagiwara M, Sonis S. Oral mucositis in patients undergoing radiation treatment for head and neck carcinoma. Cancer. 2006;106:329‐336. 10.1002/cncr.21622. 16342066

[11] Bentzen SM, Saunders MI, Dische S, Bond SJ. Radiotherapy‐related early morbidity in head and neck cancer: quantitative clinical radiobiology as deduced from the CHART trial. Radiother Oncol. 2001;60:123‐135. 10.1016/s0167-8140(01)00358-9. 11439207

[12] Harada K, Ferdous T, Yoshida H. Investigation of optimal schedule of concurrent radiotherapy with S‐1 for oral squamous cell carcinoma. Oncol Rep. 2007;18:1077‐1083. 17914556

[13] Bonner JA, Harari PM, Giralt J, et al. Radiotherapy plus cetuximab for locoregionally advanced head and neck cancer: 5‐year survival data from a phase 3 randomised trial, and relation between cetuximab‐induced rash and survival. Lancet Oncol. 2010;11:21‐28. 10.1016/S1470-2045(09)70311-0. 19897418

[14] Ohashi T, Ohnishi M, Tanahashi S, Murai M. Efficacy and toxicity of concurrent chemoradiotherapy with nedaplatin and S‐1 for head and neck cancer. Jpn J Clin Oncol. 2011;41:348‐352. 10.1093/jjco/hyq196. 21109512

[15] Common Terminology Criteria for Adverse Events version 4.0 Publish Date: May 28, 2009. https://ctep.cancer.gov/protocolDevelopment/electronic_applications/ctc.htm#ctc_40

[16] Nagatani A, Ogawa Y, Sunaga T, et al. Analysis of the risk factors for severe oral mucositis in head and neck cancer after chemoradiotherapy with S‐1. Yakugaku Zasshi. 2017;137:221‐225. 10.1248/yakushi.16-00077. 28154335

[17] Jyoti M, Jihoon L, Blythe D, Richard V, Edwin A. Smoking decreases survival in locally advanced cervical cancer treated with radiation. Am J Clin Oncol. 2018;41:295‐301. 10.1097/COC.0000000000000268. 26808259 PMC4958034

[18] Chen AM, Chen LM, Vaughan A, et al. Tobacco smoking during radiation therapy for head‐and‐neck cancer is associated with unfavorable outcomes. Int J Radiat Oncol Biol Phys. 2011;79:414‐419. 10.1016/j.ijrobp.2009.10.050. 20399030

[19] Fu KK, Pajak TF, Trotti A, et al. A radiation therapy oncology group (RTOG) phase III randomized study to compare hyperfractionation and two variants of accelerated fractionation to standard fractionation radiotherapy for head and neck squamous cell carcinomas: first report of RTOG 9003. Int J Radiat Oncol Biol Phys. 2000;48:7‐16. 10.1016/s0360-3016(00)00663-5. 10924966

